# Body Size Evolution in Burying Beetles (Staphylinidae: Silphinae: *Nicrophorus*)

**DOI:** 10.1002/ece3.73012

**Published:** 2026-02-25

**Authors:** Ashlee N. Smith, Derek S. Sikes, J. Curtis Creighton, Seth M. Bybee, Perry L. Wood, Gareth S. Powell, Mark C. Belk

**Affiliations:** ^1^ Department of Biology Brigham Young University Provo Utah USA; ^2^ University of Alaska Museum/Department of Biology & Wildlife University of Alaska Fairbanks Fairbanks Alaska USA; ^3^ Department of Biological Sciences Purdue University Northwest Hammond Indiana USA; ^4^ Monte L. Bean Museum Brigham Young University Provo Utah USA; ^5^ Department of Ecology and Evolutionary Biology University of Michigan Ann Arbor Michigan USA; ^6^ Department of Entomology and Plant Pathology North Carolina State University Raleigh North Carolina USA

**Keywords:** coleoptera, competition, disjunct size distribution, ecological character displacement, latitudinal variation, Nicrophorini, species richness, sympatry

## Abstract

Body size is an important component of burying beetle (genus *Nicrophorus*) life history, affecting competitive interactions and resource use. Currently, there is no comprehensive analysis of what drives these differences in size and how body size is distributed within the genus and across its geographic range. We used a large dataset of body size measurements and geographical data to evaluate the relative importance of phylogeny, biogeography, and ecology in explaining body size variation in burying beetles. Mean body size distribution among species is broad (4.15–10.97 mm pronotal width) and skewed, with more small and medium‐bodied species than large species. We found evidence of phylogenetic signal in the evolution of body size across the genus, although only one instance of sister species both being giants and no instances of sister species being both small. However, the phylogenetic analysis does not explain the evolution of extremes in *Nicrophorus* body size. Areas with higher species richness have a greater spread between the largest and smallest species, and body size is divergent between most sister species and more strongly so between sympatric sister species, even after correcting for phylogeny. We found evidence of rapid initial divergence in body size following speciation, which increased over time in sympatric species, but stabilized in non‐sympatric species. Smallest body sizes and highest species richness are concentrated in northern hemisphere temperate latitudes. Taken together, these results suggest character displacement by body size may be a significant factor allowing coexistence of burying beetle species; however, other mechanisms of niche partitioning are likely important contributors to coexistence. High species richness in temperate, mesic areas of the northern hemisphere may be driven by habitat and climatic suitability. We encourage further experimentation to test our proposed mechanisms of body size divergence and geographic distribution in *Nicrophorus*.

## Graphical abstract

Burying beetles (genus *Nicrophorus*) exhibit broad, skewed variation in body size, which influences carcass use and competitive interactions. Using a large dataset of morphological and geographic data, we show that body size evolution is shaped by phylogenetic history, biogeography, and ecological interactions, with divergence especially pronounced among sympatric sister species. Our findings suggest that character displacement in body size facilitates coexistence across the genus, alongside other ecological and abiotic factors.

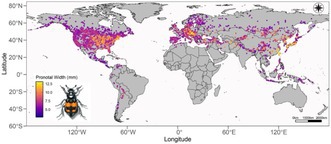

## 1 Introduction

Body size is one of the most important attributes of an animal, both evolutionarily and ecologically. Size has a predominant influence on an individual's physiology, life history, and fitness (Peters 1983; Reiss 1989; Roff 1992). It is also important in species interactions and community structure (Schoener 1974; Werner and Gilliam 1984) as well as in the process of speciation (Nagel and Schluter 1998; Schluter 2001; Miraldo and Hanski 2014).

Because of the importance of body size to the ecology and evolution of organisms, patterns of variation in body size among co‐occurring taxa have been the focus of ecological research for several years (Hutchinson 1959; Zink 2014). Body size is impacted by a complex suite of macro‐ and micro‐ecological factors as well as phylogenetic factors. Several geographic patterns of body size have been documented to predict differences in mean body size with latitude, elevation, and environmental variation (reviewed in Gaston et al. 2008). Body size is also impacted by ecological factors such as intra‐ and interspecific competition, predation, food availability, and temperature (Blanckenhorn 2000; Chown and Gaston 2010; McNab 2010). In addition, the distribution of a trait, such as body size, among closely related taxa is best assessed in a phylogenetic context (e.g., Waller and Svensson 2017). Trait variation among closely related species often reflects divergent ecological influences, whereas similarities among closely related species can result from phylogenetic relatedness (Blomberg et al. 2003; Nosil 2012). The evolution of body size results from an integration of phylogenetic, ecological, and biogeographic drivers of variation.

The genus *Nicrophorus* (commonly known as burying beetles) includes about 70 species (Sikes 2016). All members of this genus with documented life histories have elaborate biparental care behaviors and use small vertebrate carcasses as a food source for their offspring (Pukowski 1933; Scott 1998; Potticary et al. 2024), with one exception where *N. pustulatus* uses snake eggs for reproduction, but also reproduces on vertebrate carcasses (Smith et al. 2007; Quinby, Feldman, et al. 2020). The extent of parental care varies among species and ranges from facultative to obligate biparental care (Capodeanu‐Nägler et al. 2016; Jarrett et al. 2017; Potticary et al. 2024). Parental care behaviors involve burying the carcass underground (Fetherston et al. 1990), removing fur or feathers from the carcass, rolling the carcass into a ball, and applying anal and oral secretions to the ball (Trumbo et al. 2016; Potticary et al. 2024). After the young arrive on the carcass, parents guard the brood and regurgitate partially digested carrion inoculated with microbiota to their larvae as they grow (Körner et al. 2023).

Body size is an important variable strongly related to fitness in the genus *Nicrophorus*. At the individual level, body size is largely determined by brood size, which is adjusted to carcass size via filial cannibalism by the parents (Bartlett 1987; Trumbo 1990a; Creighton 2005; Damron et al. 2021). This results in a positive correlation between offspring number and carcass size (Bartlett 1987; Trumbo 1990a; Creighton 2005). The degree to which parents regulate brood size is a phenotypically plastic trait influenced by population density (Creighton 2005; Rauter et al. 2010) where females in dense populations produce smaller broods of larger offspring (Creighton 2005). Larger individual beetles are usually victorious over smaller individuals in fights for possession of both buried and unburied carcasses (Bartlett and Ashworth 1988; Müller et al. 1990; Otronen 1988; Robertson 1993; Scott and Gladstein 1993; Smith and Belk 2018). At the population level, larger individuals tend to breed on larger carcasses (Hopwood et al. 2016). At the community level, larger species are more likely to gain control of carcasses when beetles of more than one species compete for the same carcass (Otronen 1988; Wilson et al. 1984; Trumbo 1990b), and larger species tend to breed on larger carcasses (Scott 1998; Wilson et al. 1984; Trumbo 1990b; Ikeda et al. 2006). Recent work has focused on body size variation in burying beetles within species and among co‐occurring species at a local geographic scale (Rauter et al. 2010; Otronen 1988; Hopwood et al. 2016; Eggert and Sakaluk 2000; Smith 2002; Merrick and Smith 2004; Steiger 2013; Smith et al. 2014; Pilakouta et al. 2015; Trumbo and Xhihani 2015; Collard et al. 2021); however, the global evolutionary patterns of body size variation among *Nicrophorus* species and how body size is distributed across the geographic range of the genus is poorly understood. Burying beetles are well‐suited to such a large‐scale, global analysis of body size for several reasons. First, there is a high level of variation in mean body size among species in the genus *Nicrophorus* (mean pronotal width varies from 4.15 mm in *N. montivagus* to 10.97 mm in *N. concolor* ), a well‐resolved phylogeny, and geo‐ and size‐referenced occurrence dataset (Sikes and Venables 2013b), which allows for analysis of body size evolution in the context of phylogeny. Second, because burying beetles co‐occur in multi‐species communities across a variety of habitats, they enable an evaluation of ecological effects of congeners on body size. Third, because burying beetles have a broad geographic range (both longitudinally and latitudinally), we can use patterns of their distributions to understand biogeographic influences on body size.

In this study, we investigate body size variation in burying beetles in the context of phylogeny, ecology, and biogeography. Our specific objectives were: (1) to characterize the distribution and variance of mean body size of species within the genus; (2) to determine to what extent phylogeny (i.e., shared ancestry) can account for the range of variation in body size within the genus; (3) to determine how ecological competitive interactions may shape body size divergence among species when they co‐occur using phylogeny‐corrected data; and (4) to characterize biogeographic patterns in body size that may reflect relationships to latitude or other related climatic predictors. To accomplish these objectives, we evaluate one prediction related to phylogeny, two predictions related to ecology, and two predictions related to biogeography as follows: Phylogeny (1), extremes of body size are scattered across the phylogeny indicating little phylogenetic influence on evolution of extreme body size in the genus. Ecology (1), the range of body sizes (largest to smallest) will increase with increasing number of co‐occurring species in a geographic area, consistent with ecological character displacement. Ecology (2), sister species pairs that occur in sympatry will exhibit greater divergence in body size compared to sister species pairs that occur in non‐sympatry, consistent with ecological character displacement. Biogeography (1), species' mean body size is evenly distributed across latitudes indicating no differential effect of latitude‐related environmental effects on species of differing body size. Biogeography (2), species richness is significantly related to latitude indicating an influence of latitude‐related environmental effects on the number of co‐occurring species.

## 2 Methods

### 2.1 Data Compilation

**FIGURE 1:**
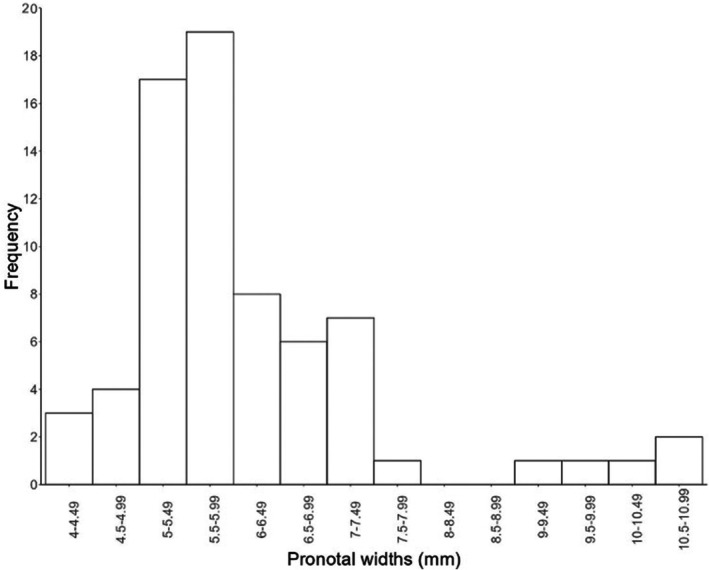
Frequency distribution of mean pronotal widths (mm) of *Nicrophorus* species.

For each of these objectives we used a dataset compiled by DSS that included 12,019 pronotal width measurements from museum specimens and 5247 pronotal widths from the scientific literature for 70 *Nicrophorus* species (see Sikes and Venables 2013b for list of museums from which specimens were borrowed). Each occurrence was georeferenced with corresponding latitude and longitude, some of which were corrected due to wrong hemispheres, from the original data of Sikes and Venables (2013a). Occurrence records from the literature, which lacked pronotal width data, were given mean values of the sex of their species, if known, or of their species, if sex was unknown. The full data set (museum + literature) was used to fill in gaps for the co‐occurring species analyses while only the museum data were used for the other analyses. Pronotal width is a standard measure of body size in burying beetles, and it scales with body size in general (Trumbo and Xhihani 2015; Smith and Belk 2018). We refer to the five species with a mean pronotal width ≥ 9 mm as “giants” because there is a clear gap in size between species with pronotal widths under 8 mm and those over 9 mm (Appendix 1, Figure 1). Although there is no gap between the smallest‐bodied and medium‐bodied species (Appendix 1, Figure 1), we refer to the seven species with mean pronotal widths under 5 mm as small‐bodied. Our analyses were all conducted using pronotal width as a continuous variable—we use these size categories only to aid in discussion of results.

### 2.2 Body Size Range Among Species and With Phylogeny

To explore the distribution of body size among species of the genus *Nicrophorus*, we calculated a mean pronotal width for each species and an overall mean and 95% confidence interval for the entire genus. We used the individual species' mean values to create a pronotal size‐frequency histogram (4.00 mm to 10.99 mm in 0.5 mm intervals). We performed a D'Agostino K‐squared test in the package ‘moments’ (Komsta and Novomestky 2015) in R (R Core Team 2013) to determine if body size (pronotal width) among all 70 *Nicrophorus* species was normally distributed. To determine if the observed distribution, including the mean, skew, and 1 mm gap were consistent with a random expectation, we compared the observed values to those obtained from two random null models. In both models we assumed that the range of sizes that are possible is bounded between 4 mm and 11 mm pronotal width.

The first model was a simple random selection of pronotal widths. We simulated a distribution of 70 mean pronotum widths (representing 70 species) by randomly selecting values between 4.00 mm and 11.00 mm, allowing two places below the decimal for each potential value. We then compiled these random values into a histogram with bin sizes of 0.5 mm (14 bins; 4–4.5, 4.5–5…10.5–11; the same as in the observed species mean pronotal width distribution). We ran the model 10,000 times and then assessed how many of the random histograms (1) exhibited a mean pronotum width less than or equal to the mean pronotum width for the entire genus, (2) exhibited a skewed distribution equal to or more extreme than that in the observed size‐frequency histogram, and (3) contained a contiguous 2‐bin gap (1.0 mm) somewhere in the internal bins of the distribution (i.e., not at the upper or lower boundaries).

The second model was a speciation model of random divergence of body size. We started with one species with pronotal width equal to the estimated ancestral size (6.67 mm pronotal width). At each step in the model (a speciation event) the species present diverged to form two new species of random size. The divergence in size was simulated as a random draw of magnitude 1 to 1.125 ratio (based on divergence measured in non‐sympatric sister species pairs) of the parent species pronotum width and a random direction (0.5 probability) either larger than or smaller than the parental species. Both “new” species were allowed to deviate in this random way. Speciation and divergence proceeded until there were 70 species. If a speciation event resulted in a species' mean pronotum width outside the 4 mm to 11 mm boundaries, the event was canceled for that lineage and the parental species size persisted until the next speciation event. The probability of speciation was set at 0.5 and the probability of extinction was set at 0.1 for each potential speciation event. We compiled the resulting species values into a histogram with bin sizes of 0.5 mm (14 bins; 4–4.5, 4.5–5…10.5–11; the same as in the observed species mean pronotal width distribution). We ran the model 1000 times and then assessed how many of the random histograms (1) exhibited a mean pronotum width less than or equal to the mean pronotum width for the entire genus, (2) exhibited a skewed distribution equal to or more extreme than that in the observed size‐frequency histogram, and (3) contained a contiguous 2‐bin gap (1.0 mm) somewhere in the internal bins of the distribution (i.e., not at the upper or lower boundaries).

We used the phylogeny in Sikes and Venables (2013a) to determine how body size is distributed across the *Nicrophorus* phylogeny. This phylogeny was estimated from four molecular markers, two protein‐coding mitochondrial (COI, COII) and two nuclear, one ribosomal and one protein‐coding (D2 region of 28S, CAD). Bayesian and Maximum Likelihood analyses were used to infer the tree and divergence dating was conducted with BEAST using Mesozoic fossil calibrations (Sikes and Venables 2013a). Their analysis included only 54 of the *Nicrophorus* species known in 2013 and does not contain one of the giant species, *N. satanas* (morphology suggests *N. satanas* is the closest relative of the species pair *N. germanicus* and *N. morio* , which are also giants [Sikes 2003]). Ancestral reconstructions of the continuous character trait pronotal width were estimated at the internal nodes using the Maximum Likelihood (ML) function fastANC in the R package phytools (Revell 2012), with the interpolation of the states along each edge using equation [2] of Felsenstein (1985). The reconstructions were then plotted using the contMap (Revell 2013) function in the R package phytools (Revell 2012). We used Pagel's λ (Pagel 1999) and Blomberg's *K* (Blomberg et al. 2003) in the phytools v.2.0.3 (Revell 2012) package in R. We estimated significance for Pagel's λ using a likelihood ratio test and for Blomberg's *K* using 10,000 randomized permutations.

### 2.3 Ecological Patterns

To determine ecological influences on body size, we made two comparisons. First, we asked if the range of body sizes (maximum to minimum) and the overall mean body size among co‐occurring species of the genus was related to the number of co‐occurring species in a given area. If body size varies more when several species coexist, this would suggest a role for competitive interactions and character displacement in body size or ecological sorting where only species that are different in body size can coexist. To estimate the number of co‐occurring species we used ArcGIS to overlay a grid of equal‐sized squares on the geographic range of the genus *Nicrophorus*. We acknowledge our geographic data suffer from collection bias with considerably more data per unit area in Europe and North America than elsewhere, especially Russia and much of interior Asia. Grid size was set at 200 km on a side or 40,000 km^2^, resulting in a total of 992 grid squares with at least one record (total number of samples used in this analysis was 12,843). Within each grid square we summed the number of species represented. Sampling effort will influence the number of co‐occurring species detected in each grid square. Clearly, the minimum number of samples required to detect the true number of co‐occurring species in a given grid square must be equal to the number of co‐occurring species. The largest number of co‐occurring species we observed among all 992 grid squares was 10. Thus, for further analysis we removed all grid squares with fewer than 10 samples, which resulted in 341 remaining grid squares.

To determine whether the number of co‐occurring species was related to the range of body sizes observed, we plotted the mean pronotal width of the largest and smallest species (and the 95% confidence intervals) represented in the grid square. Number of grid squares, mean number of samples in each grid square, and mean minimum and mean maximum pronotal widths for each number of co‐occurring species from 1 to 7+ are given in Appendix 2. To determine if the range of species sizes varied with the number of co‐occurring species, we used the overall mean pronotal width and the mean pronotal width of the largest species and the smallest species (analyzed separately) as the response variable in a general linear model (ANOVA) with number of co‐occurring species (range 1 to 7+) as a discrete predictor. We acknowledge that these data likely have some bias resulting from the tendency for researchers to focus on regions with higher burying beetle species richness resulting in potential under‐sampling of regions with lower richness. However, by removing grid squares with small sample sizes (< 10) we have somewhat ameliorated this concern. In addition, if we sample a given number of grid squares that have only one species and we compare that to the same number of grid squares that have 7 species, the second sample will have 7 times the number of species and thus may differ from the first simply because there is a larger sample size in each grid square. To determine if this larger sample size can account for the observed divergence in mean maximum and mean minimum pronotal widths we observed, we used a random null model for comparison. We simulated a distribution of 50 mean pronotum widths (representing 50 grid squares where only one species occurred) by randomly selecting values between 4.00 and 11.00 mm, allowing two places below the decimal for each potential value. We ran the same model for each number of co‐occurring species from 1 to 7, such that in the model for two species, we randomly selected 2 × 50 (100 total) mean pronotum widths, and so on until in the model for 7 co‐occurring species we randomly selected 7 × 50 (350 total) mean pronotum widths. We ran each model 1000 times, and calculated the mean, mean maximum, and mean minimum pronotal widths (and corresponding 95% confidence intervals). We compared the overall simulated means and 95% confidence intervals to that observed empirically.

Second, to determine whether there is evidence of character displacement in body size in response to potential competition between sister species, we compared mean pronotal widths of sympatric and non‐sympatric (i.e., allopatric and parapatric) sister species pairs (*n* = 19). Under a null model of no evidence of character displacement, body sizes of sister species pairs should be equal; whereas, if character displacement has occurred between sister species, we expect significantly different body sizes to evolve especially in sympatric sister species pairs. Sister species were based on the phylogeny of Sikes and Venables (2013a) and locality data came from the published literature (Appendix 3). We used two‐sample *t*‐tests to compare the body sizes of sister species pairs. In two sister species pairs, ( *N. smefarka* + *N. przewalskii* ) and ( *N. pustulatus* + *N. hispaniola* ), we used two‐sample Wilcoxon tests for our comparison because at least one of the species' body size distributions was not normally distributed. Because we conducted 19 tests, we used a Bonferroni correction to the *p*‐value that we considered as indicating a significant difference (i.e., *p* < 0.0026; 0.05/19). Because sister species pairs with greater evolutionary distance to their most recent common ancestor are expected to show greater trait divergence than sister species pairs with smaller distances, we performed a phylogenetically independent contrasts (PICs) test (Felsenstein 1985), which incorporates branch length data from the phylogeny of Sikes and Venables (2013a), for our 19 sister species comparisons. We used OpenAI's ChatGPT (version 5.1) to assist with R code and used the R (R Core Team 2013) packages ape (Paradis and Schliep 2019), phytools (Revell 2024), and geiger (Pennell et al. 2014). To assess the influence of sympatry on size divergence while accounting for phylogeny, we performed a Phylogenetic ANCOVA in R on these 19 phylogenetically independent contrasts (∣C∣) against evolutionary divergence (Sum of Branch Lengths) and Sympatric Status.

To further explore the magnitude of character displacement, we compared the ratio of pronotal widths between sister species pairs in sympatry and non‐sympatry. The ratio of pronotal widths was calculated by dividing the mean pronotal width of the larger species by the mean pronotal width of the smaller species. We analyzed the ratio of differences in pronotal widths using a *t*‐test, with the type of isolation (i.e., sympatry or non‐sympatry) as the predictor variable and the ratio of mean width (between sister species) as the response variable. All statistical analyses of sister species pairs were performed in R (R Core Team 2013).

### 2.4 Biogeographical Patterns

We determined how mean pronotal widths of species are distributed across the geographic range of the genus *Nicrophorus* by mapping the pronotal width of each observation (museum and literature data) in R. We made a scatter plot of the mean latitude of each species and the mean species' pronotal width, and we applied a regression model using the following formula: lm(mean species' pronotal width ~ mean latitude). We realize that latitude has no specific biological meaning, so we use latitude as a convenient surrogate for the multiple biological gradients that might vary with latitude. Analyses were performed in R (R Core Team 2013). To determine how the number of co‐occurring species varies with latitude we used the geographic grid structure generated above (to test for the relationship between species pronotal width and the number of co‐occurring species). We plotted the number of co‐occurring species (corresponding to color) within each grid square across the geographic distribution to give a visual illustration of the relationship with latitude.

## 3 Results

### 3.1 Body Size Variation Among Species and Phylogenetic Patterns

Mean pronotal width of the 70 *Nicrophorus* species is 6.15 mm (95% CI = 5.82–6.48 mm). Species with mean pronotal widths from 5.0 to 6.0 mm constitute 51.4% of the total number of species. Seven species (10%) have mean pronotal widths smaller than 5.0 mm, and 22 species (31.4%) have mean pronotal widths between 6.0 and 8.0 mm. No *Nicrophorus* species has a mean pronotal width between 8.0 and 9.0 mm, creating a gap in the distribution. Five species (7.1%) are exceptionally large (“giants”) and constitute the disjunct right segment of the body size distribution (Figure 1, Appendix 1). The distribution of mean pronotal width is significantly skewed; there are many more small‐ and medium‐sized species compared to larger species (untransformed, two‐sided D'Agostino test: skew = 1.8341, z = 4.9455, *p* < 0.0001; Figure 1). Under the null model of random selection, the probability of observing a mean pronotal width lower than 6.15 mm, or a skew value greater than 1.83, or a 1 mm gap in the distribution of pronotal widths was 0 out of 10,000 simulations. Under the speciation null model, the probability of observing a mean pronotal width lower than 6.15 mm was 0.233, the probability of observing a skew value greater than 1.83 was 0.000, and the probability of observing a 1 mm gap in the distribution of pronotal widths was 0.043 out of 1000 simulations.

**FIGURE 2:**
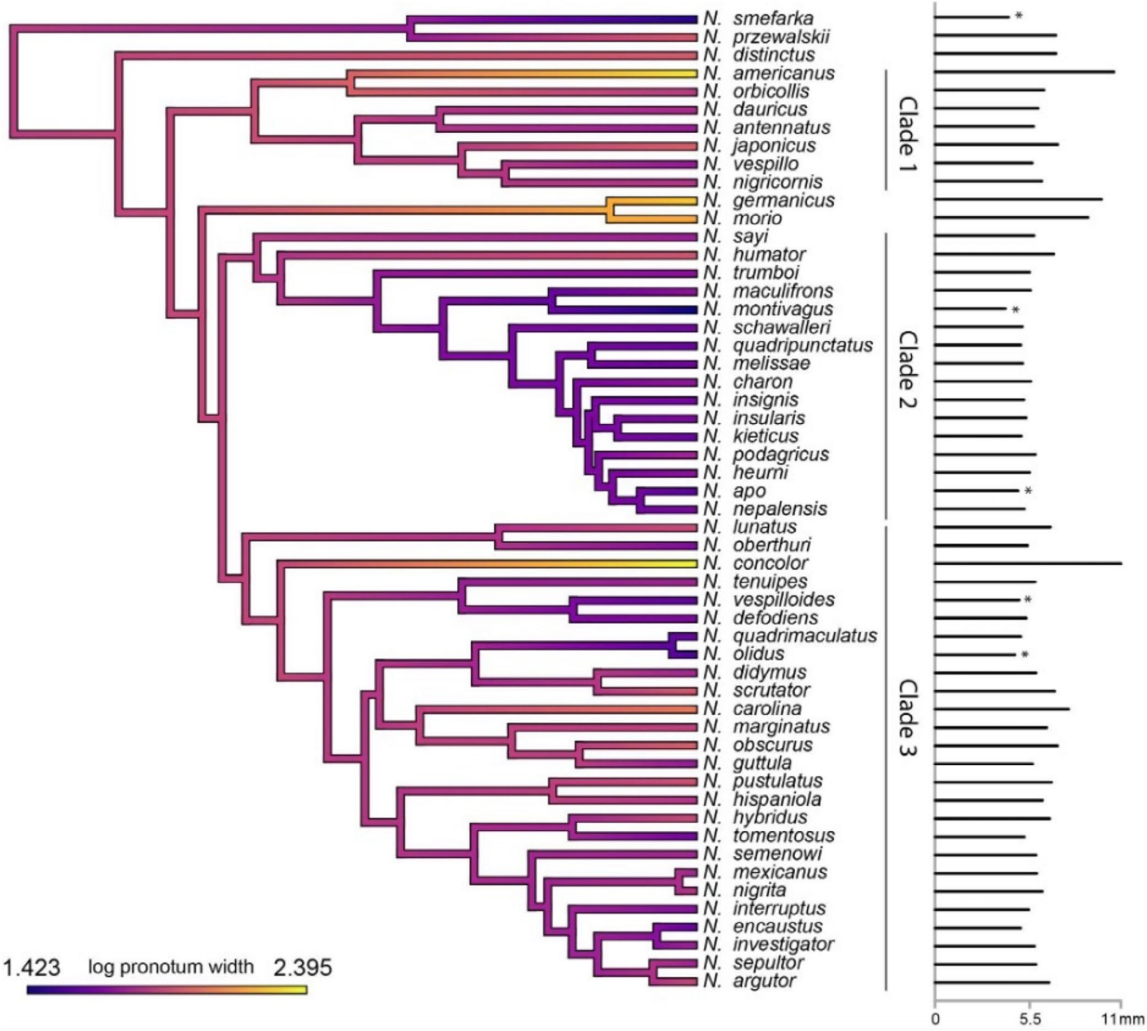
Mean burying beetle pronotal width by species for the genus *Nicrophorus* mapped onto the molecular time‐calibrated phylogeny of Sikes and Venables (2013a) using the CONTMAP function in the R package PHYTOOLS (Revell 2012). Bars on the right indicate the relative mean pronotal widths in mm and bars with an asterisk are species that have a mean pronotal width of < 5 mm. Two small species withmean pronotal widths of < 5 mm, *N. reichardti* and *N. sausai*, and one giant *N. satanas* (> 9 mm), are not included in this phylogeny.

Mean pronotal width of species mapped onto the phylogeny shows evolution of both increased and decreased size from the ancestral size (≈6.67 mm, log transformed 1.85) several times (Figure 2). The phylogeny consists of three main clades (referred to as clades 1–3 from top to bottom of Figure 2). Increases and decreases in body sizes are not concentrated in any one clade. Gigantism (≥ 9 mm) evolved at least three times and small body size (< 5 mm) evolved at least five times. Only one species pair is both giants, *N. germanicus* and *N. morio*, which form an independent, species‐poor, fourth clade outside the three main clades. Smaller‐bodied species are most common in clade 2, which lacks a giant species. In contrast, smaller‐bodied species are less common than medium‐bodied species in clades 1 and 3, which contain one giant species each. None of the five smallest‐bodied species (< 5 mm) in the phylogeny are sister species (Figure 2, Appendix 1). These nine species that exhibit extremes in body size (4 giant and 5 small) resulted from eight transitions. However, our Pagel's λ and Blomberg's *K* analyses both showed strong phylogenetic signal in evolution of body size (λ = 0.999927, *p* < 0.0001; *K* = 0.708084, *p* = 0.0025). We therefore re‐ran these analyses without these nine species of extreme body size, and this new analysis also showed strong phylogenetic signal in the evolution of body size (λ = 0.580002, *p* = 0.011; *K* = 0.691792, *p* < 0.00006).

Based on the dated phylogeny of Sikes and Venables (2013a), the three lineages of giants are descended from relatively old speciation events (mean 67.9 Ma, SD = 10.67). This is noteworthy because 81% (44/54) of the species in the phylogeny are descended from speciation events younger than 40 Ma, and the five smallest‐bodied species in the phylogeny are descended from much younger speciation events than the giants (mean 20.56 Ma, SD = 16.9; Welch Two Sample *t*‐test, *t* = 4.86, df = 5.88, *p* = 0.0029). The eight cases of sympatric sister species appear to have been reproductively isolated for longer (mean of 28.34 Ma, SD = 16.0) than the 11 cases of non‐sympatric sister species (mean of 16.0 Ma, SD = 12.4) but these means were not significantly different (Welch Two Sample *t*‐test, *t* = 1.81, df = 12.75, *p* = 0.093).

### 3.2 Ecological Effects on Body Size

**FIGURE 3:**
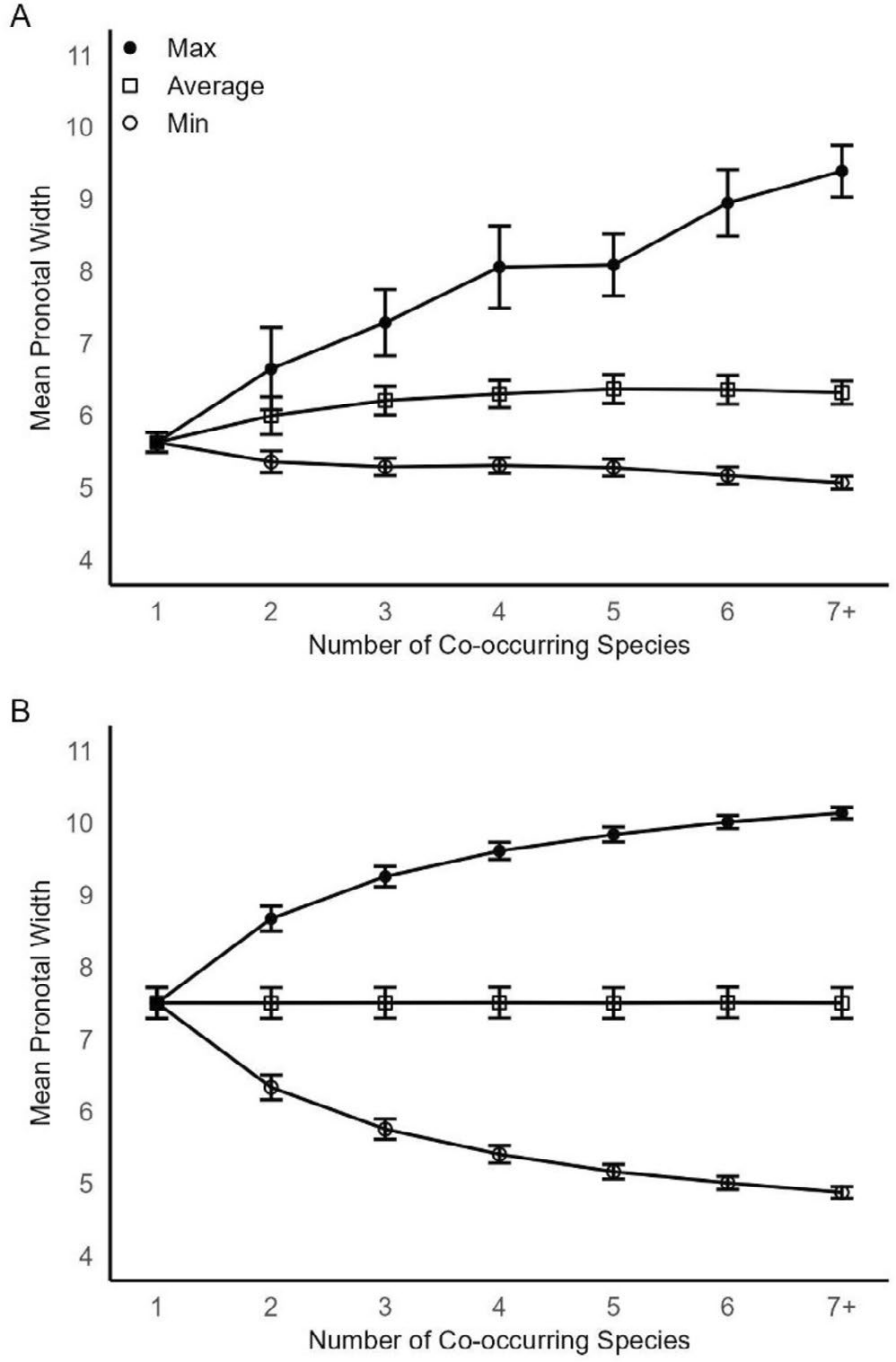
(A) Observed mean minimum pronotal width (open circles) in mm (error bars are 95% CI), mean pronotal width (open squares) in mm (error bars are 95% CI), and mean maximum pronotal width (closed circles) in mm (error bars are 95% CI) for each number of co‐occurring species from 1 to 7+ (i.e., 7–10 co‐occurring species). (B) Simulated mean minimum pronotal width (open circles) in mm (error bars are 95% CI), mean pronotal width (open squares) in mm (error bars are 95% CI), and mean maximum pronotal width (closed circles) in mm (error bars are 95% CI) for each number of co‐occurring species from 1 to 7+ (i.e., 7–10 co‐occurring species).

The number of co‐occurring species is a significant predictor of the mean pronotal width of the largest species, the smallest species, and the overall mean of all species (Table 1). In the cells that have only one species occurring, mean pronotal width is 5.62 mm (95% CI = 5.39–5.87 mm), which is significantly smaller, based on non‐overlapping confidence intervals, than the estimated ancestral size of 6.67 mm (95% CI = 5.09–8.24) and the overall mean size across all 70 species in the genus (i.e., 6.15 mm). Mean pronotal width of the largest species increases as the number of co‐occurring species increases from 1 to 7+ (Figure 3). Mean pronotal width of the smallest species decreases as the number of co‐occurring species increases, but at a slower rate compared to the maximum species size (Figure 3A). Overall mean pronotal width increases as the number of co‐occurring species increases, but only from one to three co‐occurring species. For grid cells with three or more co‐occurring species, overall mean pronotal width is relatively constant (Figure 3A). Under the null model of divergence with increasing number of co‐occurring species, the observed mean is the midpoint of the range from 4 to 11 mm pronotal width at 7.5 mm. Both the mean maximum and the mean minimum diverge from the mean symmetrically with the addition of co‐occurring species (maximum divergence equals 2.6 mm at 7+ co‐occurring species). The increment of divergence is greatest from 1 to 2 co‐occurring species (1.16 mm) and decreases consistently thereafter (0.12 mm at 6 to 7+ co‐occurring species; Figure 3B). For comparison, the observed pattern of divergence with increasing number of co‐occurring species differs from that expected under the null model of divergence with increasing sample size (Figure 3B). The observed divergence pattern is highly asymmetrical. The divergence from the mean of the observed mean maximum pronotal width (with 7+ co‐occurring species) is 3.76 mm and the divergence of the observed mean minimum pronotal width is only 0.56 mm (with 7+ co‐occurring species). Consequently, the observed divergence of maximum pronotal width is greater than that observed in the simulation; whereas the observed divergence of minimum pronotal width is much less than that observed in the simulation.

**TABLE 1:** Analysis of Variance of the maximum and minimum mean body size (pronotal width) of species as predicted by the number of co‐occurring species.

	Source	df	*F*	*p*
Maximum pronotal width	Number of co‐occurring species	6/334	30.61	< 0.0001
Mean pronotal width	Number of co‐occurring species	6/334	5.46	< 0.0001
Minimum pronotal width	Number of co‐occurring species	6/334	8.04	< 0.0001

**FIGURE 4:**
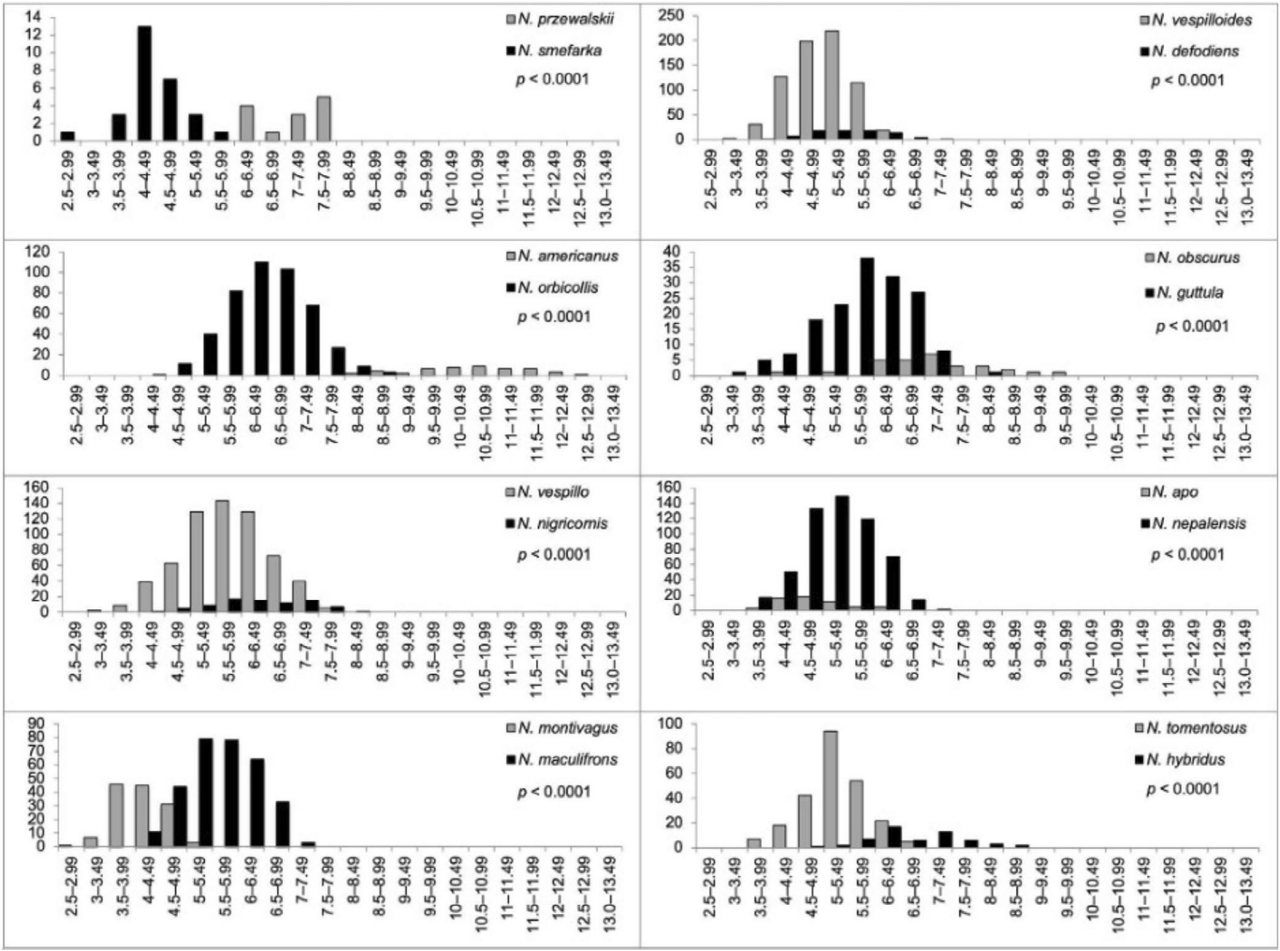
Pronotal width (mm) distributions for eight sympatric sister species pairs of *Nicrophorus*. Significance of the *t*‐test for differences of pronotal widths between species is indicated by the *p*‐value (*p*‐values < 0.0026 are considered significant).

**FIGURE 5:**
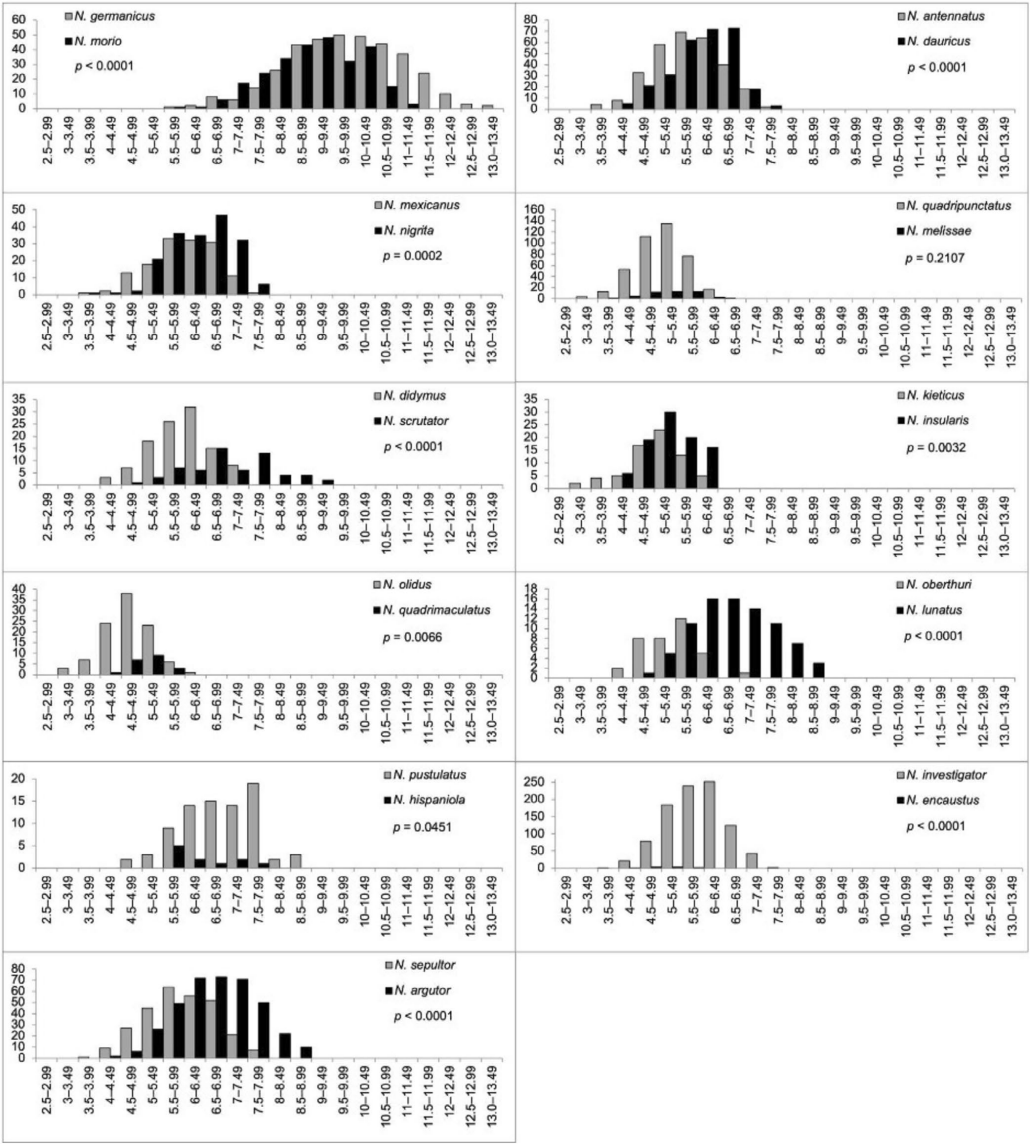
Pronotal width (mm) distributions for 11 non‐sympatric sister species pairs of *Nicrophorus*. Significance of the *t*‐test for differences of pronotal widths between species is indicated by the *p*‐value (*p*‐values < 0.0026 are considered significant).

**FIGURE 6:**
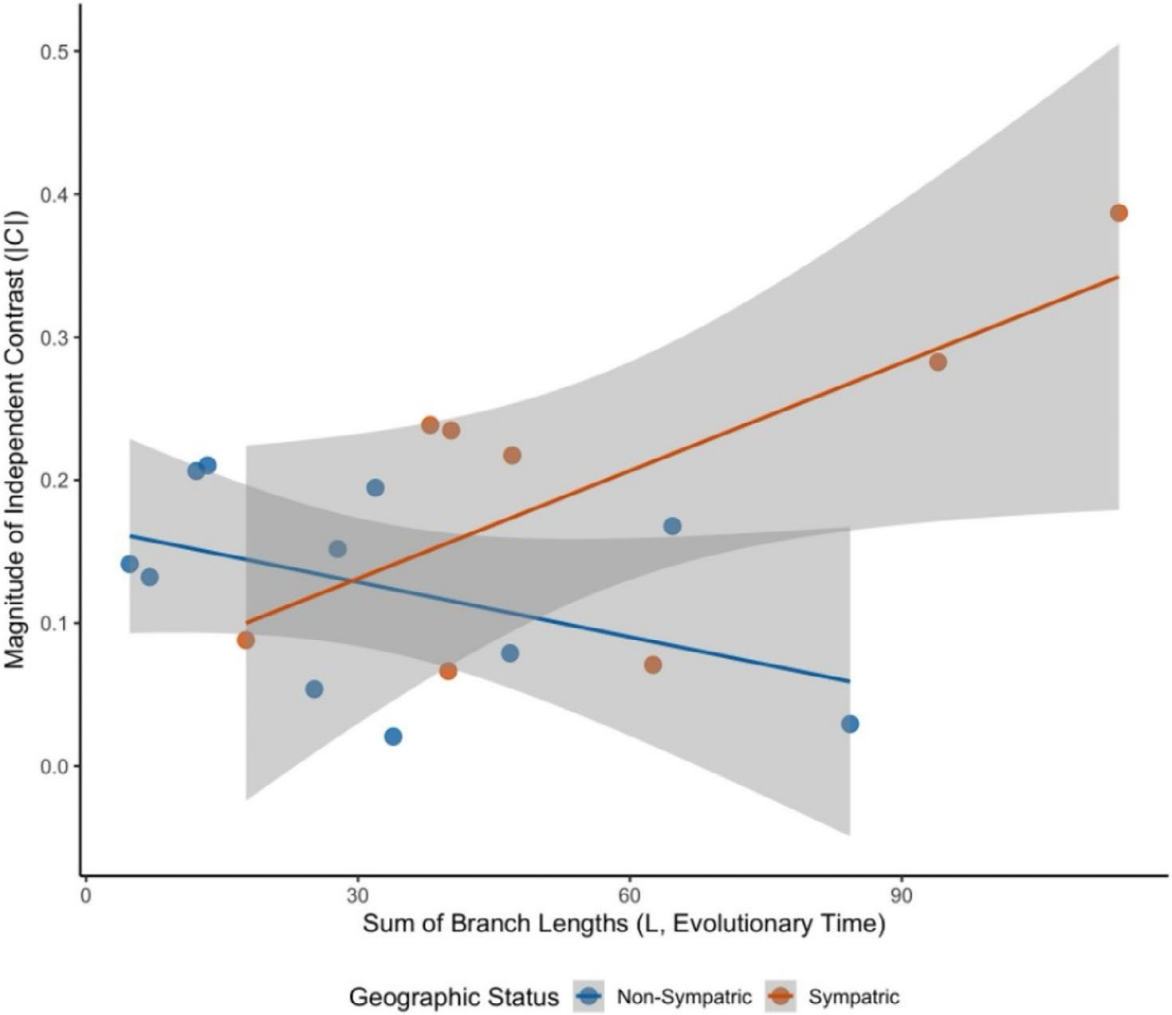
Relationship between the magnitude of size divergence and evolutionary time for 19 sister species pairs of *Nicrophorus* by sympatry status. Separate linear regression lines (with standard error shading) are fitted for sympatric (orange line) and non‐sympatric (blue line) sister species pairs.

All sympatric sister species (8 of 8 species pairs, Figure 4) and 64% of the non‐sympatric sister species (7 of 11 species pairs, Figure 5) have significantly different mean pronotal widths. Of the nineteen total *Nicrophorus* sister species pairs, 79% showed significant differences in pronotal widths (Figures 4, 5). A slight majority of sister species pairs, 58%, are non‐sympatric. Sympatric sister species differ from each other by a mean ratio of 1.31 (range = 1.08–1.64). Non‐sympatric sister species differ from each other by a mean ratio of 1.10 (range = 1.02–1.25). The ratios of body sizes between sympatric and non‐sympatric sister species were significantly different (*t* = 2.53, df = 17, *p* = 0.0223). The ratios were on average 19% higher in sympatric compared to non‐sympatric sister species. A one‐sample *t*‐test of phylogenetically independent contrasts for pronotal widths of these sister species pairs did not differ from zero (mean = −0.022, *p* = 0.62), indicating differences in sister species contrasts are not consistently biased in one direction by phylogeny, which justifies treating them as independent. The full ANCOVA model was significant (*F* = 4.716, *p* = 0.0164, Adjusted R^2^ = 0.382). This analysis revealed a significant interaction between evolutionary time and sympatric status (*p* = 0.0106), demonstrating that the rate of size divergence accumulation differs significantly based on sympatry status (Figure 6). Both groups show a significant initial divergence (shared positive intercept, *p* < 0.001). However, the slope for sympatric pairs (+0.0025) is positive and significantly different from zero (*p* = 0.01261), suggesting size divergence continues to accumulate over time in sympatry, while the slope for non‐sympatric pairs (−0.0013) is not significantly different from zero (*p* = 0.1998), suggesting size differences seen at speciation stabilizes and do not increase over time.

### 3.3 Biogeographical Patterns

**FIGURE 7:**
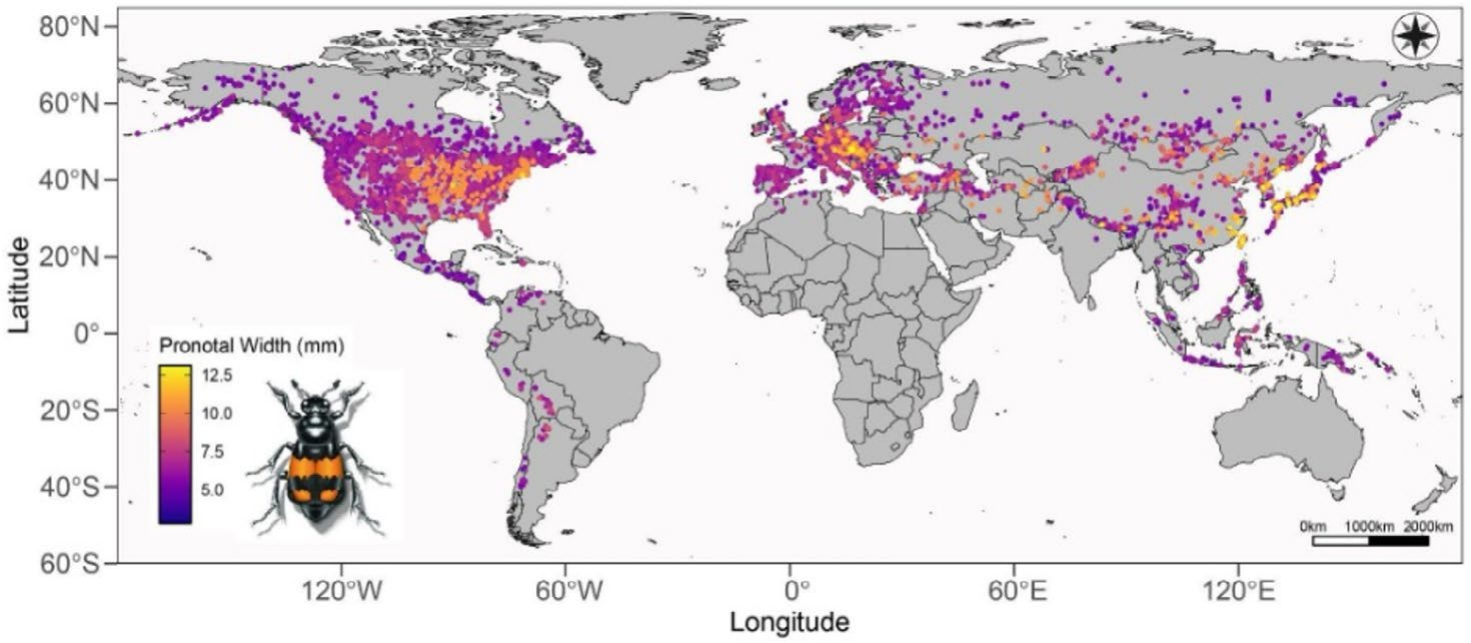
Geographic distribution of *Nicrophorus* species with each point colored on a continuous scale based on pronotal width. Lighter colors represent larger pronotal width and darker colors represent smaller pronotal width in millimeters.

**FIGURE 8:**
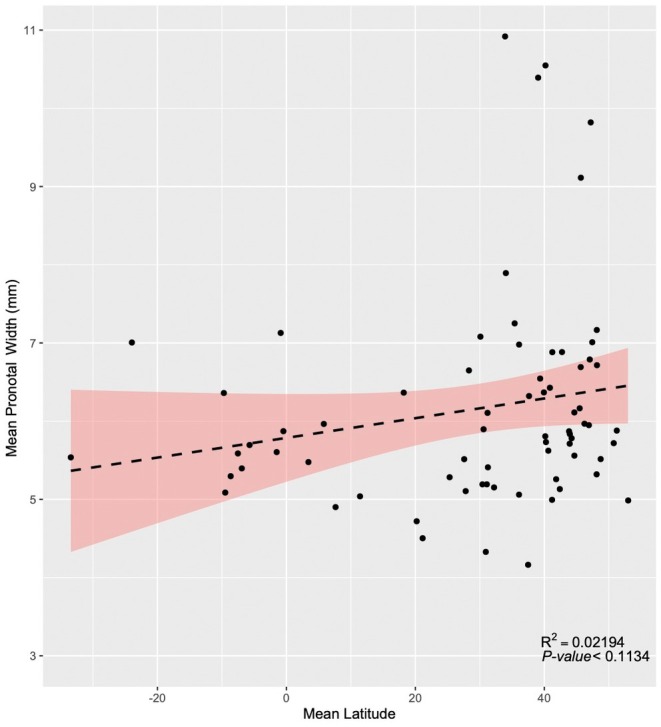
Scatter plot of the *Nicrophorus* species comparing species' mean latitude and pronotal width; eq. lm(latitude~pronotum width). Dashed line is the result of a smoothing function, and the shading is the confidence interval around the regression lines.

**FIGURE 9:**
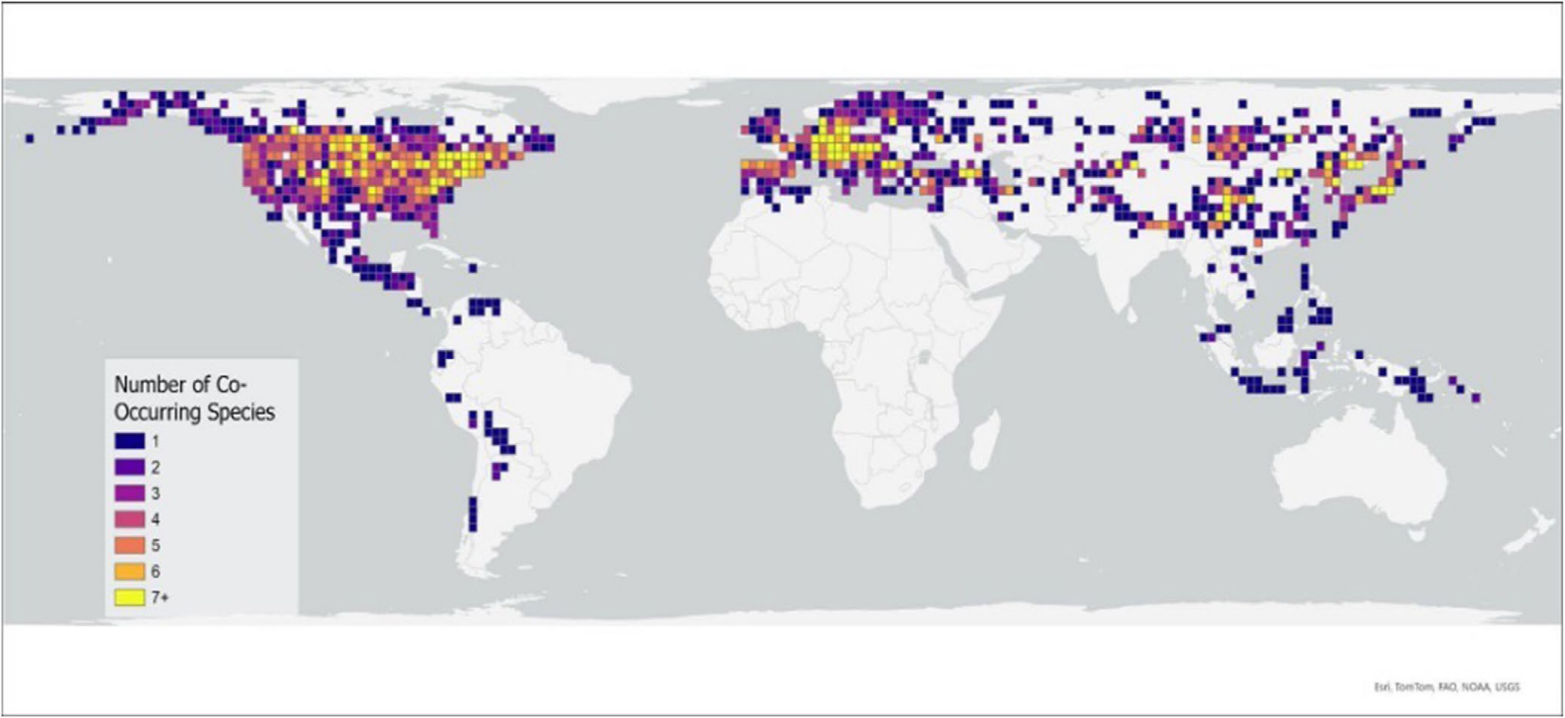
Geographic plot of number of co‐occurring species within each grid square. Grid squares measure 200 km on a side. Number of co‐occurring species ranged from 1 to 10, but grid squares with ≥ 7 co‐occurring species are combined to create sufficient sample sizes for statistical comparison. Lighter colors correspond to greater species richness.

Visual analysis of the distribution map based on body size (Figure 7) and the plot of species' mean pronotal width by latitude (Figure 8) indicates four patterns of body size distribution across the global geographic range of burying beetles. (1) The largest range in mean body sizes occurs in the northern hemisphere mid‐latitudes (30° to 50° N; Figures 7 and 8). (2) Species' mean pronotal widths and species' mean latitudes show no significant linear relationship (Figure 8). (3) Giant species occur in a restricted latitudinal range and only in the northern hemisphere (Figure 8), where the number of co‐occurring species is highest (30° to 50° N; Figure 9) and six of the seven smallest‐bodied species also occur there, but in a wider range of species' mean latitudes (20° to 53° N; Figure 8), with one southerly outlier, *N. apo* (mean 7.62° N). In contrast, medium‐sized species (pronotal widths of 5–8 mm) mainly occur in the northern hemisphere but also in the southern. They are found from 38° South to 53° North, across a much wider latitudinal range than small or giant species (Figure 8). (4) The giant species of burying beetles are not found in more arid areas such as the western United States, the Gobi Desert, or much of the Tibetan Plateau, which also have few to no burying beetles in general (Figure 7).

## 4 Discussion

Burying beetles exhibit wide variation in body size among species. Mean, minimum, and maximum body size of species of *Nicrophorus* are all large relative to other genera of Coleoptera (Stork et al. 2015). Our analysis of phylogenetic, ecological, and biogeographic influences on body size suggests an important role for all three factors in the evolution of body size of *Nicrophorus*. We evaluate the implications of phylogenetic, ecological, and biogeographic processes to the evolution of body size in the genus *Nicrophorus*.

### 4.1 Distribution of Body Size Among Species

The large range of body sizes and the relatively large mean body size within the genus *Nicrophorus* is exceptional among coleopteran genera. The lower limit of 4 mm pronotal width likely represents the minimum size that is able to process and reproduce on the smallest of vertebrate carcasses. The upper limit of 11 mm pronotal width may represent a similar boundary for use of larger vertebrate carcasses by a pair of beetles. Processing a vertebrate carcass and burying it is a costly endeavor both in time and energy (Potticary et al. 2024).

Beyond the large size range and the relatively large overall size, perhaps the most surprising feature of the distribution of body sizes among *Nicrophorus* species is the gap between the giant species and the remaining species. Our analysis suggests that the gap in species' mean pronotal width is unlikely to be a function of random processes. Both of our null models of species' size distributions generated via random processes exhibited an extremely low probability of observing an internal gap of 1 mm pronotal width in the distribution. However, individual body size varies widely within species of *Nicrophorus* (Appendix 1), and there are some individuals in our data that exhibit body sizes within this 8–9 mm pronotal width gap. Individual body size is highly labile and depends on size of the natal carcass, density of siblings on the carcass, and density of conspecifics in the environment. In some widely distributed species, mean body size might vary among local populations as a response to availability of carcass sizes and density of co‐occurring species. To unravel the interaction and implications of body size on a local scale, and to assess the importance of the “gap” in species' mean body size at the local scale, we need detailed data on the distribution of body sizes encountered over the course of the active season coupled with abundance data for all co‐occurring species in a given area. Unfortunately, although local collections of multiple species collected on the same date are available in the Sikes and Venables (2013b) data, none of those collections appear to represent the full range of species, sizes, and abundances in a given area and none represent this same level of collection over time. Thus, our estimates of overall (global) species' mean sizes are not meant to explain variation that might be encountered in a specific geographic location, but rather to explore patterns at the global level that likely result from long‐term selection on body size.

At the global level, what might account for the disjunct nature of this size distribution and the gap in species' mean pronotal width between 8 and 9 mm? In species where the size distribution of resources is closely tied to the size distribution of the consumer (e.g., large mammalian predators, gape‐limited fishes, burying beetles), gaps in the size distribution of resources can result in corresponding gaps in the size distribution of consumers. However, the distribution of body sizes in small vertebrates, the reproductive and food resource of burying beetles, is relatively continuous with no evidence of gaps in the size distribution (Ernest 2005). Persistence of burying beetles of differing sizes in a local community may be related to the abundance of vertebrate carcasses of differing sizes, not just to the distribution of carcass sizes. As a general rule, smaller vertebrates (mostly mammals and birds) will almost always be more abundant than larger vertebrates in a given area (Peters 1983). For example, in the temperate northern hemisphere, murid and cricetid rodents (with a mass of about 20–50 g) are considerably more abundant compared to small sciurids and lagomorphs (mass of about 100–300 g), the next most abundant group (Nowak 1991). Thus, the gap in mean body size distribution may be related to the necessary size of burying beetle required to process larger, but less abundant carcasses. A better understanding of how body size of the largest burying beetle species relates to their reproductive success (e.g., Belk et al. 2021) would be helpful in resolving this question.

Alternatively, substantial differences in the life history (especially lifespan), trophic position, or habitat use (aquatic versus terrestrial) among species within the same genus can lead to disjunct patterns in body size distributions. However, life history traits, habitat use, and trophic position in burying beetles show no patterns that correspond to a body size gap. Finally, disjunct geographic distributions, especially occupancy of isolated islands, can result in disjunct patterns of body size among closely related species (Trumbo and Thomas 1998). Islands represent unique environments and community assemblages that can drive rapid changes in body size. For example, many Pleistocene era mammals exhibit dramatically different body size patterns on islands compared to mainland populations (Raia and Meiri 2006). However, none of these causes of disjunct size distributions observed in other taxa seem to explain the lack of *Nicrophorus* species with mean pronotal widths between 8 and 9 mm.

One of the most prevalent characteristics of body size distributions among species is a disproportionate representation of small‐sized taxa (Gaston and Blackburn 2000; Kozłowski and Gawelczyk 2002). Although this pattern is well documented in vertebrates (reviewed in Trumbo and Thomas 1998), it remains relatively understudied in invertebrates (Waller and Svensson 2017; Rainford et al. 2016). Our results show that medium and smaller body sizes are also more common in *Nicrophorus* (Figure 1). Several hypotheses have been presented to explain such skewed data (reviewed in Kozłowski and Gawelczyk 2002). One possible explanation for long‐tailed distributions of body size is a higher rate of speciation in small species and a higher rate of extinction in large species due to their correspondingly smaller population sizes (Gould 1988; Brown and Maurer 1989). These ideas may be consistent with patterns in burying beetles because the most recent speciation events in *Nicrophorus* have resulted in smaller species (Figure 2), and at least one of the giant species, *Nicrophorus americanus*, is a United States‐federally protected species at risk of extinction (Lomolino et al. 1995). Similarly, *N. germanicus* is IUCN Red Listed as Vulnerable (Růžička and Jakubec 2017) and threatened with extinction (Schmidl et al. 2021) in the Czech Republic and Germany, respectively. To our knowledge, conservation assessments have not been done for *N. germanicus* in other countries or for the other large species of *Nicrophorus*. Mechanisms underlying the decline of these two giant species are not fully understood but are most likely tied to their use of larger‐sized carcasses for reproduction (Anderson 1982b; Kozol et al. 1988; Sikes and Raithel 2002). For example, larger carcasses are more rare than the smaller carcasses used by smaller burying beetle species. This discrepancy is magnified in fragmented habitats (Nupp and Swihart 2000). Larger carcasses are also more costly to bury and preserve, potentially reducing future reproductive opportunities (Belk et al. 2021).

Another potential explanation of the skewed distribution of body sizes in *Nicrophorus* is that burying beetle body sizes are related to the body size distribution of small vertebrates. Burying beetles partition carcasses according to body size, with smaller species exploiting smaller carcasses and larger species exploiting larger carcasses (Scott 1998; Wilson et al. 1984; Trumbo 1990b; Ikeda et al. 2006). In general, vertebrate body size distributions show an overrepresentation of small species (Blackburn and Gaston 1994a, 1994b; Sikes et al. 2024), so body size in burying beetles likely covaries with available small vertebrate carcass size. These two hypotheses (i.e., differential speciation and extinction, and size distribution of resources) are not mutually exclusive. The prevalence of small and medium body sizes in the genus *Nicrophorus* is likely a combination of both processes.

### 4.2 Phylogenetic Influence on Body Size

Although shared evolutionary history seems to explain little of the distribution of extremes in burying beetle body size, our tests for phylogenetic signal indicate that closely related species have more similar body sizes than species drawn at random. This suggests that body sizes may have been subject to evolutionary constraints that have led them to evolve according to their phylogenetic relationships. Despite this general tendency, we did not find the majority of the largest or smallest species to be close relatives. There are nine cases of extremes in body size in our analysis (Figure 2), resulting from eight independent evolutionary changes in body size. Gigantism has evolved at least three times in burying beetles (Figure 2). Only one of these cases appears to have resulted in more than one giant species—that of *N. germanicus* and *N. morio* , which are non‐sympatric sister species and the closest relatives of the giant species *N. satanas* (Sikes 2003), which is missing from the molecular phylogeny. These three giants presumably share a giant common ancestor. Supporting this assertion, we estimated the ancestor of *N. morio* and *N. germanicus* to have a pronotum width of 9.19 mm. The other two giant species, *N. americanus* and *N. concolor* , are not closely related to any still‐extant giant species (Figure 2). None of the seven smallest‐bodied species are closely related, resulting from at least five originations of small body size (two of the smallest species, *N. reichardti* and *N. sausai* , are missing from the phylogeny). Therefore, something other than phylogenetic relationships is needed to explain eight of these nine cases of extreme body size. With these nine species of extreme body size removed, our phylogenetic signal analysis remained significant. This indicates these giant and small‐bodied species are not critical to the detection of phylogenetic signal in these data, that is, the signal is primarily driven by body size variation among the species of non‐extreme size.

The sister genus to *Nicrophorus*, *Eonecrophorus* (Kurosawa 1985), known from a single specimen collected in far eastern Nepal, has a pronotal width of 4.62 mm (Sikes 2003), and the sister genus to this pair of genera, *Ptomascopus*, with three species, has a combined mean pronotal width of 4.25 mm (Sikes 2003). These close relatives of *Nicrophorus* are significantly smaller than our estimate of the pronotal width of the common ancestor of *Nicrophorus* (mean 6.67 mm; 95% CI = 5.09–8.24) and the overall mean pronotal width across all 70 *Nicrophorus* species (6.15 mm). We do not know the sister taxon of the Silphinae, but we do know that taxon is a staphylinid (Sikes et al. 2024) and that the Silphinae are much larger‐bodied than most staphylinids (Sikes et al. 2024). Although outside the focus of our investigation, these relationships suggest there was considerable evolution toward larger body size along the nicrophorine lineage that resulted in the genus *Nicrophorus* with at least five cases of later transitions to smaller body size within the genus. Size data from the Silphini (sister taxon to the Nicrophorini) and recently described Mesozoic fossil silphines (Sikes et al. 2024) would be critical to include in an analysis focused on understanding this transition from small‐bodied Staphylinidae to large‐bodied Silphinae. However, this would also ideally include the staphylinid sister‐group to the Silphinae, which remains uncertain, despite considerable phylogenetic effort (Sikes et al. 2024). Such an analysis might result in changes to our estimate of the size of the common ancestor of the genus *Nicrophorus*.

The evolutionary timing of speciation does seem to relate to the origination of giant and small‐bodied species, with the former evolving from much older speciation events than the latter, but not whether they are sympatric or otherwise. The explanation for this evolutionary timing pattern in relation to body size is unknown.

### 4.3 Ecological Influences on Patterns of Body Size

In our analysis, body size diverged asymmetrically as the number of co‐occurring species increased from 1 to 7+. In contrast, the effect of adding additional species in our simulation model resulted in symmetrical divergence of both the maximum and minimum pronotal widths. Thus, some of the observed divergence between minimum and maximum pronotal width can be explained by randomly adding species. However, the asymmetrical divergence and the magnitude of divergence in mean maximum pronotal width suggest a role for competition and resulting niche partitioning and character divergence in more diverse assemblages of burying beetles. The asymmetrical pattern of divergence, where maximum body size diverges more than would be expected by randomly adding species to a local assemblage, suggests that especially large species (i.e., giants) may only exist in highly diverse burying beetle communities.

The most well‐studied giant burying beetle is *N. americanus* , which is the largest *Nicrophorus* species in North America (Lomolino et al. 1995; Schnell et al. 2008). *Nicrophorus americanus* and its sympatric, medium‐sized sister species, *N. orbicollis*, have significant overlap in their geographic ranges, habitat preferences, diel periodicity, and breeding seasons (Lomolino et al. 1995; Lomolino and Creighton 1996; Creighton and Schnell 1998; Szalanski et al. 2000; Sikes and Raithel 2002). Thus, they are potentially direct competitors for carcasses, and this competition may have driven the evolution of gigantism in *N. americanus* . There is overlap in suitable carcass size between *N. americanus*, which prefers carcasses between 30 and 500 g, and *N. orbicollis* , which prefers carcasses between 7 and 150 g (Trumbo and Bloch 2000). In competitions for carcasses, the largest competitors of each sex gain primary access to the resource (Bartlett and Ashworth 1988; Müller et al. 1990; Otronen 1988; Smith and Belk 2018; Kozol et al. 1988; Safryn and Scott 2000; Hopwood et al. 2013; Lee et al. 2014), thus competition with other burying beetle species and resource partitioning could have driven the evolution of large body size in burying beetles. *Nicrophorus americanus* and *N. concolor* ( *N. concolor* is the largest species in Asia) both co‐occur with as many as seven other burying beetle species over parts of their ranges (Ikeda et al. 2006; Lomolino and Creighton 1996). For *N. americanus* , before its decline during the 20th century, co‐occurring species included *N. carolina* , *N* *. marginatus* , *N. orbicollis, N. pustulatus, N* *. sayi* , *N. tomentosus* , and *N. hebes* (Lomolino and Creighton 1996; Sikes et al. 2016). For *N. concolor*, co‐occurring species include *N. investigator* , *N. japonicus* , *N. maculifrons* , *N. nepalensis* , *N. montivagus* , *N. quadripunctatus* , and *N. tenuipes* (Ikeda et al. 2006; Sikes et al. 2006). Large numbers of co‐occurring burying beetle species are likely to drive intense competition for carcasses. In particular, we would expect direct competition between the largest co‐occurring species, including *N. concolor* and *N. japonicus* (see Appendix 1 for mean body sizes), which have the same habitat preferences (Ikeda et al. 2006; Růžička et al. 2002; Sikes et al. 2002).

Thus, competitive pressures may have driven the evolution of large body size in all three originations of gigantism in *Nicrophorus*. None of the five giant *Nicrophorus* species co‐occur. *Nicrophorus germanicus* and *N. morio* are parapatric sister species in the western and eastern portions of their respective ranges (Lee et al. 2014; Sikes et al. 2016), but where they co‐occur, they do not directly compete due to different habitat preferences (Scott 1998; Růžička et al. 2002; Dekeirsschieter et al. 2011). There appears to be an upper limit to burying beetle size, which may be related to competition with vertebrate scavengers over larger carcasses (Sikes and Raithel 2002).

Environmental conditions might also affect the distribution of large burying beetle species, which occur across Europe, Asia, and eastern North America, but not west of the American Rocky Mountains. The largest North American species, *N. americanus* , is known to prefer grasslands or mature forests with deep soils (Lomolino and Creighton 1996; Bedick et al. 1999), which may be related to the depth at which they bury carcasses (20–68 cm [Ferrari 2014]). Thus, the relatively shallow, rocky soils in the western United States may be unsuitable. These same niche requirements might also explain why the distributions of giant burying beetle species are restricted to geographic regions with high burying beetle species richness, that is, these may have exceptional environmental conditions and seasonal resource abundance to support diversity and speciation.

The evolution of smaller body size is likely also a mechanism to reduce competition. Burying beetles experience competition for carcasses with other insects and decomposers (Scott 1998; Sun et al. 2014; Mashaly et al. 2020), so using smaller carcasses that take less time to prepare and maintain may be beneficial, and smaller species tend to use smaller carcasses. The minimum limit to body size in burying beetles is more restricted than the maximum body size (at least compared to ancestral or current mean body size). Although vertebrate body size distributions tend to be dominated by small‐bodied species (Blackburn and Gaston 1998), even the smallest vertebrates are as large as or larger than burying beetles. As such, requirements for vertebrate carcass manipulation and preparation may limit the minimum size of burying beetles. For example, burial depth of carcasses may vary with body size in burying beetles (Potticary et al. 2024). Smaller species cannot bury as deeply as larger species. Burial depth may vary in response to surface temperature as well as the competitive environment (Potticary et al. 2024). Thus, surface temperature and competitive environment may interact to limit the minimum size of burying beetles that may be viable in a given environment.

Competition is an important driver of speciation (Schluter 2000a, 2000b; Moen and Wiens 2009), and it has been linked to speciation in other invertebrates such as dung beetles and amphipods (Miraldo and Hanski 2014; Jeffrey et al. 2017). Body size is a major factor in niche differentiation among closely related species (Wilson 1975). Burying beetles engage in intense inter‐ and intraspecific competition for access to carcasses, and the largest individuals generally control access to the resource (Bartlett and Ashworth 1988; Müller et al. 1990; Otronen 1988; Safryn and Scott 2000; Hopwood et al. 2013; Lee et al. 2014). Thus, competition could influence diversification in this group through resource partitioning according to body size. In our data, the mean pronotal widths of sister species were significantly different from each other in 15 of 19 species pairs (Figures 5 and 6).

We also found that body size differences were greater in sympatric sister species than in non‐sympatric sister species and that sympatry between sister species pairs was less common (8 of 19) than non‐sympatry (11 of 19). Our ANCOVA analysis with Phylogenetic Independent Contrasts on body size of sister species revealed that the slope for sympatric pairs is significantly steeper than that for non‐sympatric pairs (Figure 6). This indicates that while divergence is initiated quickly in all pairs, the magnitude of size difference continues to accumulate over time in species pairs that are sympatric while not doing so in sister species pairs that are non‐sympatric. This finding provides phylogenetically corrected evidence consistent with a model of character displacement (Brown and Wilson 1956), where size divergence is accelerated or sustained by interspecific competition in the areas of sympatry. The non‐zero intercept rejects a gradual model and implies that a substantial portion of the evolutionary divergence in size occurs almost immediately upon, or shortly after, the split of sister species. This is potentially a signature of rapid, early divergence driven by factors like selection on reproductive isolation, strong genetic drift, and/or ecological niche shifts that occurred near the species boundary.

Traits such as body size have been proposed as possible drivers of diversification because changes in these traits may have a significant impact on ecological opportunity and allow shifts in niche availability (Losos 2010), and the rate of body size evolution is correlated with diversification on a macroevolutionary scale (Ricklefs 2004; Rabosky et al. 2013). Rapid shifts in size have been noted alongside increased rates of diversification in several adaptive radiations (Schluter 1993; Harmon et al. 2010). Douglas (1987) showed that greater dissimilarities in body size within a clade resulted in lower competition coefficients, which may also reduce the level of competition among burying beetle species. Sympatric sister species would be in direct competition with each other, so greater differences in body size may make it more possible for them to co‐occur because larger species tend to breed on larger carcasses and smaller species breed on smaller carcasses (Scott 1998; Wilson et al. 1984; Trumbo 1990b; Ikeda et al. 2006), thus reducing competition among species through the use of different carcass sizes. A recent review of burying beetle ecology pointed out that there are few field studies on natural carcass choice (Potticary et al. 2024). However, two previous field studies showed that *N. defodiens* (a relatively small species) faced more intense competition from larger species such as *N. orbicollis* and *N. sayi* on large carcasses than small carcasses (Trumbo 1990a), and large *N. vespilloides* were more likely to abandon small carcasses than small *N. vespilloides* were to abandon large carcasses (Hopwood et al. 2016), which is likely due to the low reproductive value of small carcasses. This preference for carcasses that match body size might contribute to the divergence in body size seen between sister species. Additionally, Trumbo and Thomas (1998) documented competitive release in the form of a significantly larger body size for *N. defodiens* , a relatively small medium‐bodied species, on an isolated island lacking its normal congener competitors. Competitive release toward larger body size was also found by Sun et al. (2020) for *N. vespilloides*, a small‐bodied species, in an isolated woodland with significantly reduced competitive pressure from its larger‐bodied congeners. These two examples of reduced character displacement in non‐sympatry, combined with our results showing patterns of size distribution and size disparity covarying with number of co‐occurring species and greater size disparity between sympatric sister species, are consistent with the hypothesis that body size variation in burying beetles is in part a product of ecological character displacement (Brown and Wilson 1956; Dayan and Simberloff 2005; Pfennig and Pfennig 2009).

Burying beetles can reduce competition via character displacement or niche partitioning on axes other than body size. Co‐occurring species of *Nicrophorus* are known to differ in habitat use (grasslands versus forest), in active season (spring and autumn versus summer), in diel activity patterns (diurnal versus crepuscular or nocturnal), and other potential niche axes (Wilson et al. 1984; Anderson 1982a; Cook et al. 2019; Burke et al. 2023). A careful evaluation of our overall results suggests that competition among species based on body size and corresponding resource use is likely a dominant driver of evolution of body size in the genus *Nicrophorus*; however, competition is not likely the only influence on the patterns of variation in body size we documented.

Only 54 of the 70 extant burying beetle species are included in the phylogeny of Sikes and Venables (2013a). Therefore, some of the sister species relationships included in our analysis may differ with a larger and more complete taxon sampling. For example, *N. vespilloides* and *N. defodiens* are recovered as sister species in Sikes and Venables (2013a) (Figure 2), but recent molecular data found that *N. hebes*, a species absent from Sikes and Venables (2013a), is the sister species to *N. vespilloides* (Sikes et al. 2016). These two species are parapatric, or possibly allopatric, and have different habitat preferences (Sikes et al. 2016; Anderson 1982a; Burke et al. 2023). However, we predict that the pattern of significant differences between sizes of sister species will remain, since body size is an important part of burying beetle community structure (Scott 1998; Wilson et al. 1984; Trumbo 1990b; Ikeda et al. 2006), and possibly a driver of speciation within the group.

### 4.4 Biogeographical Patterns of Body Size

Generally, species richness of animals and plants increases toward the equator (Rosenzweig 1995; Gaston 1996; Brown and Lomolino 1998). However, patterns of biodiversity in insects do not always follow this trend (Gaston 1996; Kouki 1999; Skillen et al. 2000). *Nicrophorus* species occur predominantly in the temperate northern hemisphere, with relatively few species occurring in South America and the tropics (Figure 7). The genus is hypothesized to have originated in temperate Asia (Sikes and Venables 2013a; Hatch 1927; Peck and Anderson 1985) with eight dispersal events between the Palearctic and Nearctic (Sikes and Venables 2013a). The predominantly temperate pattern observed in burying beetles may be related, in part, to burying beetle thermal tolerances. Burying beetles show the highest activity rates at moderate temperatures (Merrick and Smith 2004; Jacques et al. 2009; Quinby, Belk, and Creighton 2020), which may be due to an inability to function at low temperatures and risk of desiccation at high temperatures (Bedick et al. 2006). There is also a higher cost of reproduction at higher temperatures where bacteria and fungi can more rapidly colonize and grow on a carcass (Jacques et al. 2009). In support of this hypothesis, *Nicrophorus* species in tropical areas are generally found at higher elevations (Sikes and Venables 2013a; Sikes et al. 2006) and are uncommon in arid environments (Sikes 2016). Additionally, burying beetles have a narrow range of temperatures in which they will reproduce (Quinby, Belk, and Creighton 2020; Keller et al. 2021). Therefore, the distribution of burying beetles is likely constrained by colder temperatures to the north and high temperatures and humidity resulting in competition with bacteria, fungi, ants, flies, and other necrophages (Scott 1998; Ferrari 2014; Sun et al. 2014) which increase toward the equator (Stork 2018; Schultheiss et al. 2022).

One curious pattern in the distribution of *Nicrophorus* is the relatively few southern hemisphere species (Figure 7). Five species occur in central and South America, none in sub‐Saharan Africa or Australia, and eight in tropical southeast Asia. This pattern of lineages being predominant in either northern or southern hemispheres is common across many taxa (e.g., Giribet et al. 2012; Wood et al. 2013). Such distributions are usually explained by the evolution or diversification of taxa after the split of Pangea into the northern supercontinent of Laurasia and the southern supercontinent of Gondwanaland, resulting in a vicariant pattern of distribution in one hemisphere or another (Jordan et al. 2016). In a general sense, this probably explains the predominance of *Nicrophorus* in the northern hemisphere. However, the lack of *Nicrophorus* species in sub‐Saharan Africa and the relatively few species in South America and southeast Asia suggests the existence of ecological barriers to dispersal and colonization in addition to the ancient, but no longer existing, water barrier (Sikes 2005).

Although the dataset we have used for this global analysis is extensive as far as specimens held in museums and reported in the literature, there are some obvious geographic gaps in our data that may constrain our conclusions. Large gaps in sampling are located in northern Canada, eastern Europe, Russia, and China (Figure 7). Some of these gaps may represent lack of sampling while others might be areas of low diversity or abundance of burying beetles. For example, the Gobi Desert and the Tibetan Plateau may include relatively few species and low abundances of burying beetles because they are extremely dry and/or cold environments. In contrast, based on the species richness observed in more suitable environments, in well sampled areas like North America and western Europe, much of Russia and China is likely to have diverse and abundant burying beetle communities. We encourage increased sampling and collection in poorly documented areas, and we suggest that this may yield new areas of diversity, new distribution records of species, and potentially new species of burying beetles. In spite of this possibility, the large size and global coverage of our current dataset provides a global perspective of the evolution of body size in *Nicrophorus*, and it seems unlikely that such additional data would lead to changes in the overall patterns we have identified.

## 5 Conclusion

This global investigation indicates that burying beetle body size variation appears to be driven primarily by competitive interactions that intensify with increasing species richness, resulting in divergence in preferred carcass sizes and corresponding beetle body sizes. Niche partitioning via divergences in phenology, diel periodicity, and habitat preferences (e.g., Wilson et al. 1984; Anderson 1982a; Burke et al. 2023) has been shown to be additional key parameters, alongside body size divergence, allowing burying beetle species co‐occurrence.

We found evidence of phylogenetic signal in body size suggesting that the largest species share larger than average close relatives and the smallest species share smaller than average close relatives. The overall relatively large size of species of *Nicrophorus* may be related to the use of small vertebrate carcasses for reproduction. The wide range of variation in size among species of *Nicrophorus*, especially where many species co‐occur, may be a result of evolutionary outcomes of competitive interactions. The largest body sizes and the highest species diversity are both concentrated in temperate latitudes of the northern hemisphere suggesting potential barriers (environmental or ecological) to dispersal into the southern hemisphere.

In addition to the individual effects of phylogeny, ecology, and biogeography, these three factors are likely to play coordinated roles in the evolution of body size in the genus. Biogeographic history determines regional lineages and the sequence of colonization events, while phylogeny tracks ancestral trait values in a lineage or region and limits subsequent evolutionary change. Ecological context mediates how lineages partition resources, respond to competitors, and exploit available niches, often generating divergent selective pressures even among closely related taxa, and those ecological parameters are tightly linked to biogeography. In a system such as burying beetles where access to discrete, limiting resources is a key axis of competition, such interactions can promote ecological specialization and shifts in body size, a trait tightly linked to performance, competitive ability, and reproductive output. Our results allow us to assess the intertwined effects of biogeographical history, phylogenetic influences, and ecological factors on one of the most important physical characteristics of nicrophorine species: body size.

## Author Contributions

**Ashlee N. Smith:** conceptualization (equal), data curation (equal), formal analysis (equal), investigation (equal), project administration (equal), writing – original draft (lead), writing – review and editing (equal). **Derek S. Sikes:** conceptualization (equal), data curation (lead), formal analysis (equal), funding acquisition (equal), investigation (equal), resources (equal), supervision (equal), writing – review and editing (equal). **J. Curtis Creighton:** conceptualization (equal), data curation (equal), methodology (equal), writing – review and editing (equal). **Seth M. Bybee:** conceptualization (equal), formal analysis (equal), methodology (equal), supervision (equal), writing – review and editing (equal). **Perry L. Wood Jr:** data curation (equal), formal analysis (equal), methodology (equal), writing – review and editing (equal). **Gareth S. Powell:** data curation (equal), formal analysis (equal), investigation (equal), visualization (equal), writing – review and editing (equal). **Mark C. Belk:** conceptualization (equal), formal analysis (equal), investigation (equal), methodology (equal), project administration (equal), resources (equal), supervision (equal), visualization (equal), writing – original draft (equal), writing – review and editing (equal).

## Funding

Field, museum, and lab work for this project was supported in part by an MCZ Ernst Mayr grant, a NSERC Discovery grant, a National Science Foundation Grant (DEB‐9981381), a University of Connecticut Research Council grant, and a National Geographic Society grant.

## Conflicts of Interest

The authors declare no conflicts of interest.

## Supporting information


**Data S1:** ece373012‐sup‐0001‐Apppendices.zip.

## Data Availability

The data that support the findings of this study are available at https://doi.org/10.5061/dryad.c2fqz61h6.
