# Supplementary material for: Body Size Evolution in Burying Beetles (Staphylinidae: Silphinae: Nicrophorus)
Source: Ecol Evol. 2026 Feb 25;16(3):e73012. doi: 10.1002/ece3.73012 (PMC12936394; doi:10.1002/ece3.73012)
Supplement: Supplementary file 1 — Data S1: ece373012‐sup‐0001‐Apppendices.zip. [file ECE3-16-e73012-s001.zip › ECE3_73012_External.docx]

Appendix 3. Data sources to support the categorization of *Nicrophorus* sister species as sympatric or non-sympatric..

| **Category** | **Sister Species Pair** | **Sources** |
| --- | --- | --- |
| Sympatric |  |  |
|  | *N. smefarka* and *N. przewalski* | Růžička et al. 2002; Sikes et al. 2002 |
|  | *N. americanus* and *N. orbicollis* | Lomolino and Creighton 1996; Trumbo and Bloch 2000; Kozol et al. 2000; Bedick et al. 1999; Anderson 1981; Peck and Kaulbars 1987; Walker and Hoback 2007 |
|  | *N. vespillo* and *N. nigricornis* | Růžička et al. 2002; Dekeirsschieter et al. 2011; Ghahari and Háva 2015 |
|  | *N. maculifrons* and *N. montivagus* | Ikeda et al. 2006; Sikes et al. 2006 |
|  | *N. vespilloides* and *N. defodiens* | Trumbo and Thomas 1998; Trumbo and Bloch 2000; Peck and Kaulbars 1987; Beninger and Peck 1982 |
|  | *N. obscurus* and *N. guttula* | Peck and Kaulbars 1987; Walker and Hoback 2007 |
|  | *N. apo* and *N. nepalensis* | Sikes et al. 2006; Růžička et al. 2002 |
|  | *N. hybridus* and *N. tomentosus* | Peck and Kaulbars 1987; Sikes et al. 2008 |
| Non-sympatric |  |  |
|  | *N. germanicus* and *N. morio* | Růžička et al. 2002; Sikes et al. 2002; Dekeirsschieter et al. 2011 |
|  | *N. mexicanus* and *N. nigrita* | Peck and Anderson 1985; Peck and Kaulbars 1987; Márquez et al. 2015 |
|  | *N. didymus* and *N. scrutator* | Peck and Anderson 1985; Sikes and Chaboo 2015 |
|  | *N. olidus* and *N. quadrimaculatus* | Peck and Anderson 1985; Márquez et al. 2015 |
|  | *N. dauricus* and *N. antennatus* | Růžička et al. 2002; Tezcan and Háva 2001; Guéorguiev and Růžička 2002 |
|  | *N. quadripunctatus* and *N. melissae* | Sikes et al. 2006; Růžička et al. 2011 |
|  | *N. insularis* and *N. kieticus* | Sikes et al. 2006; Peck 2001; Sikes and Mousseau 2013 |
|  | *N. oberthuri* and *N. lunatus* | Růžička et al. 2002; Sikes et al. 2002 |
|  | *N. pustulatus* and *N. hispaniola* | Peck and Kaulbars 1987; Sikes and Peck 2000 |
|  | *N. encaustus* and *N. investigator* | Peck and Kaulbars 1987; Sikes et al. 2008; Růžička et al. 2011 |
|  | *N. argutor* and *N. sepultor* | Růžička et al. 2002; Dekeirsschieter et al. 2011; Sikes et al. 2008 |

**References**

Anderson, R. S. 1981. “The Biology and Distribution of Silphidae and Agyrtidae of Canada and Alaska (Insecta: Coleoptera).” M.Sc. Thesis, Carleton University.

Beninger, C. W., and S. B. Peck. 1992. “Temporal and Spatial Patterns of Resource Use Among *Nicrophorus* Carrion Beetles (Coleoptera: Silphidae) in a Sphagnum Bog and Adjacent Forest Near Ottawa, Canada.” *Canadian Entomologist* **124**, no.1: 79–86.

Ghahari, H., and J. Háva. 2015. “An Annotated Checklist of the Iranian Carrion Beetles (Coleoptera: Staphylinoidea: Silphidae).” *Linzer Biologische Beiträge* **47**, no. 2: 1501–1511.

Guéorguiev, B., and J. Růžička. 2002. “Check List of Bulgarian Carrion Beetles (Coleoptera: Silphidae).” *Historia Naturalis Bulgarica* **15**: 89–112.

Márquez, J., J. Escoto‐Rocha, and I. Goyenechea. 2015. “Distribution Patterns of the Mexican Species of Carrion Beetles (Coleoptera: Silphidae).” *Coleopterists Bulletin* **69**, no. 4: 813–823.

Peck, S. B. 2001. “Review of the Carrion Beetles of Australia and New Guinea (Coleoptera: Silphidae).” *Australian Journal of Entomology* **40**, no. 2: 93–101.

Peck, S. B., and M. M. Kaulbars. 1987. “A Synopsis of the Distribution and Bionomics of the Carrion Beetles (Coleoptera: Silphidae) of the Conterminous United States.” *Proceedings of the Entomological Society of Ontario* **118**: 47–81.

Růžička, J., H. Šípková, and J. Schneider. 2011. “Notes on Carrion Beetles (Coleoptera: Silphidae) From India.” *Klapalekiana* **47**: 239–245.

Sikes, D. S., and C. S. Chaboo. 2015. “Beetles (Coleoptera) of Peru: A Survey of the Families. Silphidae”. *Journal of the Kansas Entomological Society* **88**, no. 2: 184–185.

Sikes, D. S., and S. B. Peck. 2000. “Description of *Nicrophorus hispaniola*, New Species, From Hispaniola (Coleoptera: Silphidae) and a Key to the Species of *Nicrophorus* of the New World.” *Annals of the Entomological Society of America* **93**, no. 3: 391–397.

Sikes, D. S., and T. Mousseau. 2013. “Description of *Nicrophorus efferens*, New Species, From Bougainville Island (Coleoptera, Silphidae, Nicrophorinae).” *ZooKeys* **311**: 83–93.

Sikes, D. S., S. M. Vamosi, S. T. Trumbo, M. Ricketts, and C. Venables. 2008. “Molecular Systematics and Biogeography of *Nicrophorus* in Part—The *investigator* Species Group (Coleoptera: Silphidae) Using Mixture Model MCMC.” *Molecular Phylogenetics and Evolution* **48**, no. 2: 646–666.

Tezcan, S., and J. Háva. 2001. “Notes on the Pitfall Trap Collected Carrion Beetles (Coleoptera, Silphidae) in Ecological Cherry Orchards in İzmir and Manisa Provinces of Turkey.” *Ziraat Fakültesi Dergisi* **38**, no. 1: 33–38.

Walker, T. L., and W. W. Hoback. 2007. “Effects of Invasive Eastern Redcedar on Capture Rates of *Nicrophorus americanus* and Other Silphidae.” *Environmental Entomology* **36**, no. 2: 297–307.
