# Supplementary material for: Body Size Evolution in Burying Beetles (Staphylinidae: Silphinae: Nicrophorus)
Source: Ecol Evol. 2026 Feb 25;16(3):e73012. doi: 10.1002/ece3.73012 (PMC12936394; doi:10.1002/ece3.73012)
Supplement: Supplementary file 1 — Data S1: ece373012‐sup‐0001‐Apppendices.zip. [file ECE3-16-e73012-s001.zip › Appendix 2.docx]

Appendix 2. Number of grid squares, mean (SE) number of samples per grid square, mean minimum pronotal width in mm (SE), mean pronotal width in mm (SE), and mean maximum pronotal width in mm (SE) for each number of co-occurring species from 1 to 7+ (i.e., 7-10 co-occurring species).

| **Number co-occurring species** | **Grid squares** | **Mean number of samples (SE)** | **Mean minimum size (SE)** | **Mean size (SE)** | **Mean maximum size (SE)** |
| --- | --- | --- | --- | --- | --- |
| 1 | 35 | 26.3 (3.22) | 5.63 (0.07) | 5.63 (0.12) | 5.63 (0.27) |
| 2 | 30 | 30.5 (5.89) | 5.36 (0.08) | 6.00 (0.13) | 6.65 (0.29) |
| 3 | 46 | 25.3 (3.28) | 5.29 (0.06) | 6.21 (0.11) | 7.29 (0.23) |
| 4 | 55 | 26.0 (2.72) | 5.31 (0.06) | 6.30 (0.10) | 8.06 (0.21) |
| 5 | 51 | 21.2 (1.46) | 5.28 (0.06) | 6.37 (0.10) | 8.09 (0.22) |
| 6 | 48 | 37.3 (6.32) | 5.17 (0.06) | 6.36 (0.10) | 8.95 (0.23) |
| 7–10 | 76 | 45.2 (4.41) | 5.07 (0.05) | 6.32 (0.08) | 9.39 (0.18) |
