# Supplementary material for: Body Size Evolution in Burying Beetles (Staphylinidae: Silphinae: Nicrophorus)
Source: Ecol Evol. 2026 Feb 25;16(3):e73012. doi: 10.1002/ece3.73012 (PMC12936394; doi:10.1002/ece3.73012)
Supplement: Supplementary file 1 — Data S1: ece373012‐sup‐0001‐Apppendices.zip. [file ECE3-16-e73012-s001.zip › Appendix 1.docx]

Appendix 1. Mean pronotal width, range of pronotal widths in the sample, and number of specimens measured (museum specimens only) for 70 burying beetle species sorted by size.

| **Species** | **Mean Pronotal**  **Width (mm)** | **Range (mm)** | **Sample Size** |
| --- | --- | --- | --- |
| *N. concolor* | 10.97 | 7.95–13.64 | 332 |
| *N. americanus* | 10.56 | 8.02–12.51 | 50 |
| *N. satanas* | 10.46 | 8.49–12.11 | 37 |
| *N. germanicus* | 9.83 | 5.99–13.19 | 366 |
| *N. morio* | 9.03 | 5.94–11.34 | 266 |
| *N. carolina* | 7.88 | 5.23–10.47 | 136 |
| *N. japonicus* | 7.24 | 5.09–10.05 | 183 |
| *N. obscurus* | 7.23 | 4.38–9.52 | 29 |
| *N. distinctus* | 7.14 | 5.17–8.77 | 38 |
| *N. przewalskii* | 7.08 | 5.8–7.9 | 15 |
| *N. scrutator* | 7.06 | 4.96–9.22 | 61 |
| *N. validus* | 7.06 | 5.75–8.64 | 9 |
| *N. humator* | 7.00 | 4.01–9.32 | 673 |
| *N. pustulatus* | 6.88 | 4.63–8.83 | 81 |
| *N. lunatus* | 6.81 | 4.78–8.71 | 84 |
| *N. hybridus* | 6.77 | 4.8–8.95 | 58 |
| *N. argutor* | 6.73 | 4.29–8.97 | 381 |
| *N. marginatus* | 6.59 | 4.29–9.11 | 216 |
| *N. ussuriensis* | 6.56 | 5.61–7.3 | 8 |
| *N. orbicollis* | 6.43 | 4.49–8.68 | 454 |
| *N. hispaniola* | 6.34 | 5.5–7.55 | 11 |
| *N. nigrita* | 6.33 | 3.88–7.69 | 181 |
| *N. nigricornis* | 6.30 | 4.31–8.02 | 82 |
| *N. vestigator* | 6.23 | 3.26–8.38 | 294 |
| *N. dauricus* | 6.09 | 4.08–7.87 | 285 |
| *N. mexicanus* | 6.02 | 3.86–7.96 | 141 |
| *N. reticulatus* | 6.00 | 4.87–6.6 | 4 |
| *N. sepultor* | 5.96 | 3.98–7.78 | 282 |
| *N. semenowi* | 5.96 | 4.96–6.88 | 29 |
| *N. didymus* | 5.96 | 4–7.48 | 109 |
| *N. podagricus* | 5.92 | 4.35–7.35 | 112 |
| *N. tenuipes* | 5.91 | 3.64–7.21 | 201 |
| *N. mongolicus* | 5.90 | 3.98–7.23 | 117 |
| *N. investigator* | 5.87 | 3.85–7.89 | 945 |
| *N. sayi* | 5.84 | 4.02–7.7 | 24 |
| *N. antennatus* | 5.82 | 3.84–7.58 | 296 |
| *N. guttula* | 5.76 | 3.35–8.17 | 160 |
| *N. vespillo* | 5.74 | 3.41–7.96 | 632 |
| *N. basalis* | 5.72 | 3.78–7.41 | 166 |
| *N. sepulchralis* | 5.70 | 4.22–7.17 | 73 |
| *N. efferens* | 5.70 | 5.02–6.42 | 6 |
| *N. charon* | 5.65 | 4.7–7 | 18 |
| *N. maculifrons* | 5.64 | 4–7.39 | 312 |
| *N. heurni* | 5.58 | 4.24–6.87 | 143 |
| *N. trumboi* | 5.58 | 4.56–6.35 | 17 |
| *N. interruptus* | 5.53 | 2.93–7.59 | 651 |
| *N. herscheli* | 5.48 | 4.66–6.54 | 9 |
| *N. oberthuri* | 5.46 | 4.47–7.02 | 36 |
| *N. chilensis* | 5.44 | 4.22–6.3 | 14 |
| *N. defodiens* | 5.39 | 3.74–7.05 | 83 |
| *N. insularis* | 5.36 | 4.23–6.45 | 96 |
| *N. hebes* | 5.36 | 3.5–6.77 | 420 |
| *N. tomentosus* | 5.28 | 3.61–6.93 | 242 |
| *N. nepalensis* | 5.27 | 3.59–7.07 | 555 |
| *N. insignis* | 5.25 | 4.31–6.42 | 18 |
| *N. sinensis* | 5.21 | 3.84–6.63 | 68 |
| *N. quadraticollis* | 5.19 | 4.06–6.57 | 23 |
| *N. melissae* | 5.18 | 3.66–6.26 | 47 |
| *N. schawalleri* | 5.16 | 4.15–6.49 | 77 |
| *N. encaustus* | 5.15 | 4.36–6.21 | 12 |
| *N. kieticus* | 5.09 | 3.4–6.43 | 69 |
| *N. quadripunctatus* | 5.06 | 3.4–6.77 | 412 |
| *N. quadrimaculatus* | 5.06 | 4.41–5.64 | 20 |
| *N. vespilloides* | 4.97 | 3.09–6.38 | 713 |
| *N. apo* | 4.90 | 3.57–6.35 | 58 |
| *N. reichardti* | 4.74 | 3.91–5.61 | 9 |
| *N. olidus* | 4.71 | 3.38–6.18 | 102 |
| *N. sausai* | 4.47 | 3.28–5.2 | 6 |
| *N. smefarka* | 4.34 | 2.8–5.5 | 28 |
| *N. montivagus* | 4.15 | 2.96–5.21 | 133 |
